# Artificial intelligence versus manual screening for the detection of diabetic retinopathy: a comparative systematic review and meta-analysis

**DOI:** 10.3389/fmed.2025.1519768

**Published:** 2025-05-07

**Authors:** Hasan Nawaz Tahir, Naseer Ullah, Mursala Tahir, Inbaraj Susai Domnic, Ramaprabha Prabhakar, Semmal Syed Meerasa, Ahmed Ibrahim AbdElneam, Shahnawaz Tahir, Yousaf Ali

**Affiliations:** ^1^Department of Community Medicine, College of Medicine, Dwadimi, Shaqra University, Shaqra, Saudi Arabia; ^2^Department of Community Medicine, Khyber Medical College Peshawar, Peshawar, Pakistan; ^3^Department of Community Medicine, Liaquat National Hospital and Medical College, Jinnah Sindh Medical University, Karachi, Pakistan; ^4^Department of Pharmacology, College of Medicine, Dwadimi, Shaqra University, Shaqra, Saudi Arabia; ^5^Department of Physiology, College of Medicine, Shaqra University, Shaqra, Saudi Arabia; ^6^Departments of Clinical Biochemistry and Basic Medical Sciences, College of Medicine, Dwadimi, Shaqra University, Shaqra, Saudi Arabia; ^7^Molecular Genetics and Enzymology Department, Human Genetics and Genome Research Institute, National Research Center, Dokki, Cairo, Egypt; ^8^Department of Gastroenterology, Dow University of Health Sciences, Karachi, Pakistan

**Keywords:** diabetic retinopathy, screening, artificial intelligence, deep learning, manual screening, automated detection

## Abstract

**Background:**

Diabetic retinopathy is one of the leading causes of blindness globally, among individuals with diabetes mellitus. Early detection through screening can help in preventing disease progression. In recent advancements artificial Intelligence assisted screening has emerged as an alternative to traditional manual screening methods. This diagnostic test accuracy (DTA) review aims to compare the sensitivity and specificity of AI versus manual screening for detecting diabetic retinopathy, focusing on both dilated and un-dilated eyes.

**Methods:**

A systematic review and meta-analysis were conducted for comparison of AI vs. manual screening of diabetic retinopathy using 25 observational (cross sectional, validation and cohort) studies with total images of 613,690 used for screening published between January 2015 and December 2024. Outcomes of the study was sensitivity, and specificity. Risk of bias was assessed using the QUADAS-2 tool for validation studies, the AXIS tool for cross-sectional studies, and the Newcastle-Ottawa Scale for cohort studies.

**Results:**

The results of this meta-analysis showed that for un-dilated eyes, AI screening showed pooled sensitivity of 0.90 [95% CI: 0.85–0.94] and pooled specificity of 0.94 [95% CI: 0.91–0.96] while manual screening shows pooled sensitivity of 0.79 [95% CI: 0.60–0.91] and pooled specificity of 0.99 [95% CI: 0.98–0.99]. For dilated eyes the pooled sensitivity of AI screening is 0.95 [95% CI: 0.91–0.97] and pooled specificity is 0.87 [95% CI: 0.79–0.92], while manual screening sensitivity is 0.90 [95% CI: 0.87–0.92] and specificity is 0.99 [95% CI: 0.99–1.00]. These data show comparable sensitivities and specificities of AI and manual screening, with AI performing better in sensitivity.

**Conclusion:**

AI-assisted screening for diabetic retinopathy shows comparable sensitivity and specificity compared to manual screening. These results suggest that AI can be a reliable alternative in clinical settings, with increased early detection rates and reducing the burden on ophthalmologists. Further research is needed to validate these findings.

**Systematic review registration:**

https://www.crd.york.ac.uk/PROSPERO/home, CRD42024596611.

## Introduction

Diabetic Retinopathy (DR) is one of the most prevalent microvascular complications of diabetes, characterized by damage to the retina due to prolonged hyperglycemia. It remains a leading cause of blindness globally, particularly among working-age adults. The World Health Organization (WHO) estimates that over 422 million people worldwide have diabetes (1), with approximately 103.12 million adult individuals affected by diabetic retinopathy and 160.50 million by 2045 (2). In advanced stages, untreated DR can lead to severe vision impairment and blindness. According to a 2023 global report on vision by the WHO report globally distance vision impairment or blindness from diabetic retinopathy are 3.9 million (3). Early detection and timely treatment can significantly reduce the risk of vision loss, but widespread screening remains a challenge, particularly in low-resource settings.

Screening for diabetic retinopathy has traditionally been performed through manual methods, including fundus photography, direct ophthalmoscopy, mydriatic and non mydriatic retinal photography, slit lamp microscopy, and retinal video recording conducted by trained ophthalmologists. However, these methods are often time-consuming and require specialized equipment and personnel, limiting their availability in certain regions (4). Recent technological advancements have led to the development of automated screening methods using artificial intelligence (AI). AI-based algorithms, particularly deep learning models, can analyze retinal images and detect signs of DR with comparable sensitivity and specificity to human graders. These systems have the potential to increase screening efficiency, reduce costs, and provide access to screening in underserved populations. AI has been recognized for its ability to identify DR and classify the severity of the condition, making it a valuable tool in large-scale screening programs.

There are few systematic reviews and meta-analyses which have evaluated the performance of AI-based systems for DR screening. Meta-analysis reported high sensitivity and specificity for AI algorithms (5–8). Another review (9) supported these findings but highlighted the variability in performance. However there is no review on comparison of AI vs. manual method to clarify the role of AI in different screening contexts, particularly in comparison to manual methods.

This Review aims to evaluate the performance of AI versus manual screening in DR detection. We systematically review the sensitivity and specificity of AI and manual methods, with a focus on both dilated and un-dilated eye conditions.

## Methods

### Search strategy

We conducted a literature search for AI and manual screening methods of diabetic retinopathy using PubMed and Google Scholar to identify relevant studies published between January 2015 to September 2024 and a second search was done in Feb 2025 which added 13 studies to included studies which become 25 included studies. Search strategy contain mesh terms and keywords which included “diabetic retinopathy,” “artificial intelligence,” “deep learning,” “manual screening,” and “automated detection.” Only English language articles were included if they show AI-based or manual-based screening methods for DR detection and reported sensitivity and specificity outcomes.

### Inclusion criteria

Studies were included if they were observational or validation and evaluated AI algorithms or manual screening for DR with patients aged 15 to 90 years diagnosed with DR and reported sensitivity and specificity outcomes for either dilated or un-dilated eye conditions. Studies were excluded if they did not report the outcomes of interest (specificity and sensitivity), the author of the studies did not respond or if the full text were not available.

### Study selection

**Figure 1:**
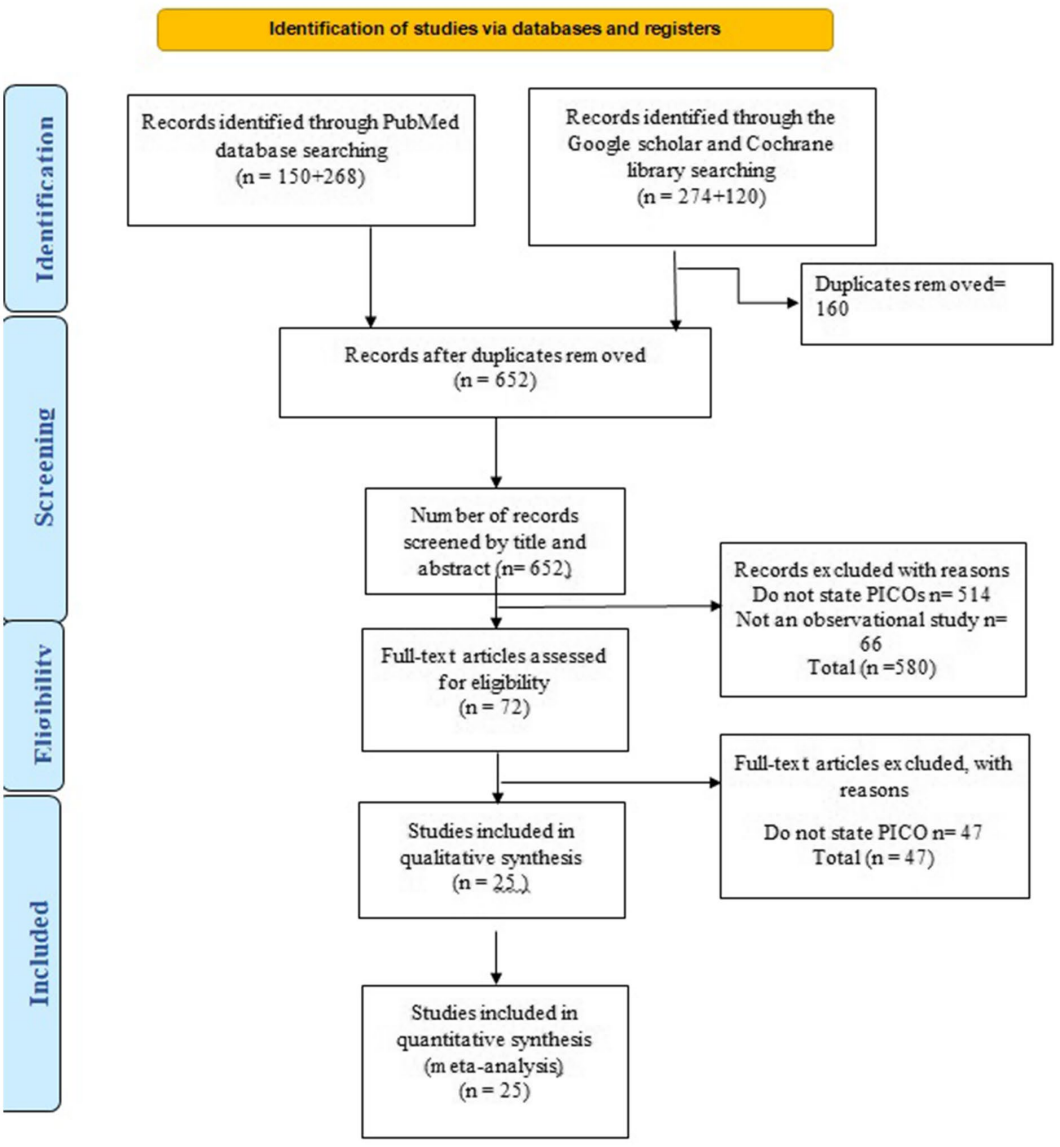
PRISMA flow diagram for included studies.

Initially two independent reviewers screened the articles by titles and abstracts. Once the articles met the inclusion criteria or were uncertain than full texts were obtained for those. The same reviewers then independently assessed the full texts. Discrepancies were resolved through discussion or, if needed, consultation with a third reviewer. PRISMA flow diagram was used for documentation of selection process Figure 1.

### Quality assessment

Each study was assessed for quality by two independent reviewers to evaluate selection bias, outcome/exposure assessment bias, follow-up bias, measurement bias, sample representativeness, reporting bias, index test bias, reference standard bias, flow and timing bias, and ethical considerations bias was evaluated. Three different tools QUADAS-2, AXIS tool, and Newcastle-Ottawa scale was used according to type of studies (validation study, cross-sectional and cohort respectively) to evaluate risk of bias, which were used for strength of evidence of meta-analysis results.

### Data extraction

Sensitivity and specificity data for AI and manual screening methods were extracted using a standardized data collection form for dilated or un-dilated eyes. Extracted information included study characteristics such as first author, country, number of participants, number of images, age of participants, comparison to human grader, photographic protocol, reference standard and outcomes of interest like sensitivity, and specificity. Two reviewers independently extracted data to minimize bias, by consensus or consulting a third reviewer disagreements were resolved. The information was initially entered into Excel tables and then transferred to Review Manager 5.4 and R-software for analysis. The risk of bias was assessed using the Newcastle-Ottawa scale for cohort studies, the AXIS tool for cross-sectional studies, and the QUADAS-2 tool for validation studies.

## Results

### Study characteristics

A total of 25 studies met the inclusion criteria of this review which evaluated Artificial intelligence based screening and manual screening for diabetic retinopathy. Twelve studies reported images of un-dilated eyes screened by AI-based or manual methods, while 14 studies show dilated eyes images screened by AI-based and manual methods. Twelve out of 25 studies were prospective (10–21), and 13 were retrospective design (22–34).

The range of sample size is from 54 to 5,738 in 19 studies with total participants of 29,358 while six studies did not mentioned number of participants but only images, 613,690 images in 25 studies were used for screening process, in a broad geographic range of settings (out patients, hospital, community based and nationwide survey) and populations. The details are given in Table 1.

**Table 1:** Characteristics of included studies.

Study	Country	Study setting	No. of images	No. of participants	Prospective	Compared to human graders	Photographic protocol	Reference standard
Ting et al. 2017 (25)	Singapore	Community-based and clinic-based populations	225,302	Not mentioned	No	Yes	2 fields images, Mydriasis	Grading by a retinal specialist (>5 years’ experience in conducting diabetic retinopathy assessment)
Sosale et al. 2020 (15)	India	Outpatient	618	297	Yes	Yes	3-fields dilated retinal imaging, Mydriasis	Adjudicated diagnosis of the two fellowship-trained vitreoretinal specialists
Surya et al. 2023 (16)	India	Outpatient	1,234	1,085	Yes	Yes	5 fields imaging, No Mydriasis	Diagnosis made by the specialist ophthalmologists
Piatti et al. 2024 (13)	Italy	Outpatient	602	598	Yes	Yes	2 field imaging, Mydriasis	Classification of the retinal images by the human ophthalmologist grader
Sedova et al. 2022 (14)	Austria	Outpatient	113	54	Yes	Yes	45-degree, 2 fields imaging, No Mydriasis	Manual grading of images by retina specialists
Ipp 2021 (10)	United states	Outpatient	4,004	893	Yes	Yes	4-wide field imaging for no Mydriasis and 2 fields imaging No Mydriasis	Grading of 4-wide-field stereoscopic dilated fundus photographs by the WFPRC
Tokuda et al. 2022 (17)	Japan	Inpatient	69	70	Yes	No	45-degree, no mydriasis	Grading of the fundus images by three retinal experts according to the ICDRS scale
Acharyya et al. 2024 (22)	India	Outpatient	1,783	Not mentioned	No	Yes	45-degree, no mydriasis	Consensus of three blinded vitreoretinal specialists, with an arbitrator resolving any disagreements.
Arenas-Cavalli et al. 2022 (23)	Chile	Outpatient	1,142	1,123	No	Yes	45-degree, 2 fields, variable for case to case	assessment performed remotely by a clinical ophthalmologist.
Li et al. 2022 (11)	China	Hospital-based study	1,464	1,147	Yes	Yes	45-degree, no mydriasis	Grading of the retinal fundus images by a certified retinal specialist with more than 12 years of experience, who used the 5-point (ICDRS) scale to assign grades
Limwattanayingyong et al. 2020 (24)	Thailand	Nationwide screening program	11,148	5,738	No	Yes	45-degree, 1 field, no mydriasis	Grading of the retinal photographs by a panel of three IRS
Lupidi et al. 2023 (12)	Italy	Outpatient	831	251	Yes	Yes	50-degree, 1 field, no mydriasis	Fundus biomicroscopic examination by an experienced retina specialist
González-Gonzalo et al. 2020 (26)	Sweden	Dataset	600	288	No	Yes	45-degree field, no mdriasis	Certified ophthalmologist with over 12 years of experience
Lin et al. 2018 (27)	United states	Dataset	33,000		No	no	not mentioned	Well-trained clinicians according to the International Clinical Diabetic Retinopathy scale
Li et al. 2019 (28)	China	Hospital-based study	19,233	5,278	No	Yes	Inner circle of retina	Expert committee of three senior ophthalmologists
Soto-Pedre et al. 2015 (18)	Spain	Dataset	10,556	5,278	Yes	Yes	45-degree field, mdriasis	One retinal specialist
Hansen et al. 2015 (29)	Kenya	Community-based	6,788	3,460	No	Yes	2 field, mydraisis	Moorfields Eye Hospitals Reading Centre in the UK
Rajalakshmi et al. 2018 (19)	India	Hospital-based study	2,408	301	Yes	Yes	45-degree field, mdriasis	Ophthalmologists (retina specialists)
Gargeya and Leng 2017 (30)	United states	Dataset	75,137	Not mentioned	No	Yes	inner retinal circle	Panel of human retinal specialists
Wang et al. 2018 (20)	India	Outpatient	1,661	383	Yes	Yes	non-steered central image, mydriasis	Certified diabetic retinopathy (DR) graders at the Doheny Image Reading Center (DIRC)
Abràmoff et al. 2016 (31)	United states	Dataset	1,748	874	No	Yes	45-degree field, mdriasis	Three US Board certified retinal specialists
Zhang et al. 2019 (32)	China	Hospital-based study	13,767	1,872	No	Yes	45-degree field, mdriasis	One retinal specialist with over 27 years of experience and two ophthalmologists with over 5 years of experience
Li et al. 2018 (21)	China and Australia	Hospital-based study	106,244	Not mentioned	Yes	Yes	45-degree field, mdriasis and non mydraisis	Panel of ophthalmologists
Zhang et al. 2022 (33)	China	Dataset	92,894	Not mentioned	No	Yes	Fundus images	Ophthalmologist used international grading system for diabetic retinopathy
Kumar et al. 2016 (34)	India	Hospital-based study	1,344	368	No	Yes	50-degree field, mdriasis	Panel of expert ophthalmologists at the Regional Institute of Ophthalmology

WFPRC, Wisconsin Fundus Photograph Reading Center; IRS, international retina specialists; ICDRS, International Clinical Diabetic Retinopathy Severity scale. ^a^These are the external datasets for which accuracy estimates were included in the meta-analysis; datasets used for training and internal validation were not included. b. “Compared to human graders” refers to whether retinal images were graded and compared with the results provided by AI with human graders. c. Where specified the mydriatic or non-mydriatic imaging protocols were followed depending on the study setting, with multiple fields captured. d. For certain studies, the primary reference standard was provided by expert ophthalmologists or retinal specialists with a minimum of 5 years’ experience in diabetic retinopathy assessment, though in some cases, decisions were made through consensus from multiple specialists or reading centers. e. External validation of these studies was conducted in clinical settings such as hospital-based, outpatient, or community-based screening programs, as specified.

### Test accuracy

The diagnostic accuracy of AI-based diabetic retinopathy (DR) screening compared to manual methods shows that, in dilated eyes, the SROC curves shows wider confidence intervals of specificities across the included studies, indicating variability in diagnostic performance.

Un-dilated eye screening tends to achieve high sensitivity and specificity values with most of the studies reporting sensitivity and specificity of more than 0.90. This suggests a reliable ability of AI algorithms to correctly identify DR in un-dilated eye examinations. The studies generally cluster around the upper-left corner of the plot, indicating strong diagnostic performance with low rates of false positives and false negatives.

**Figure 2:**
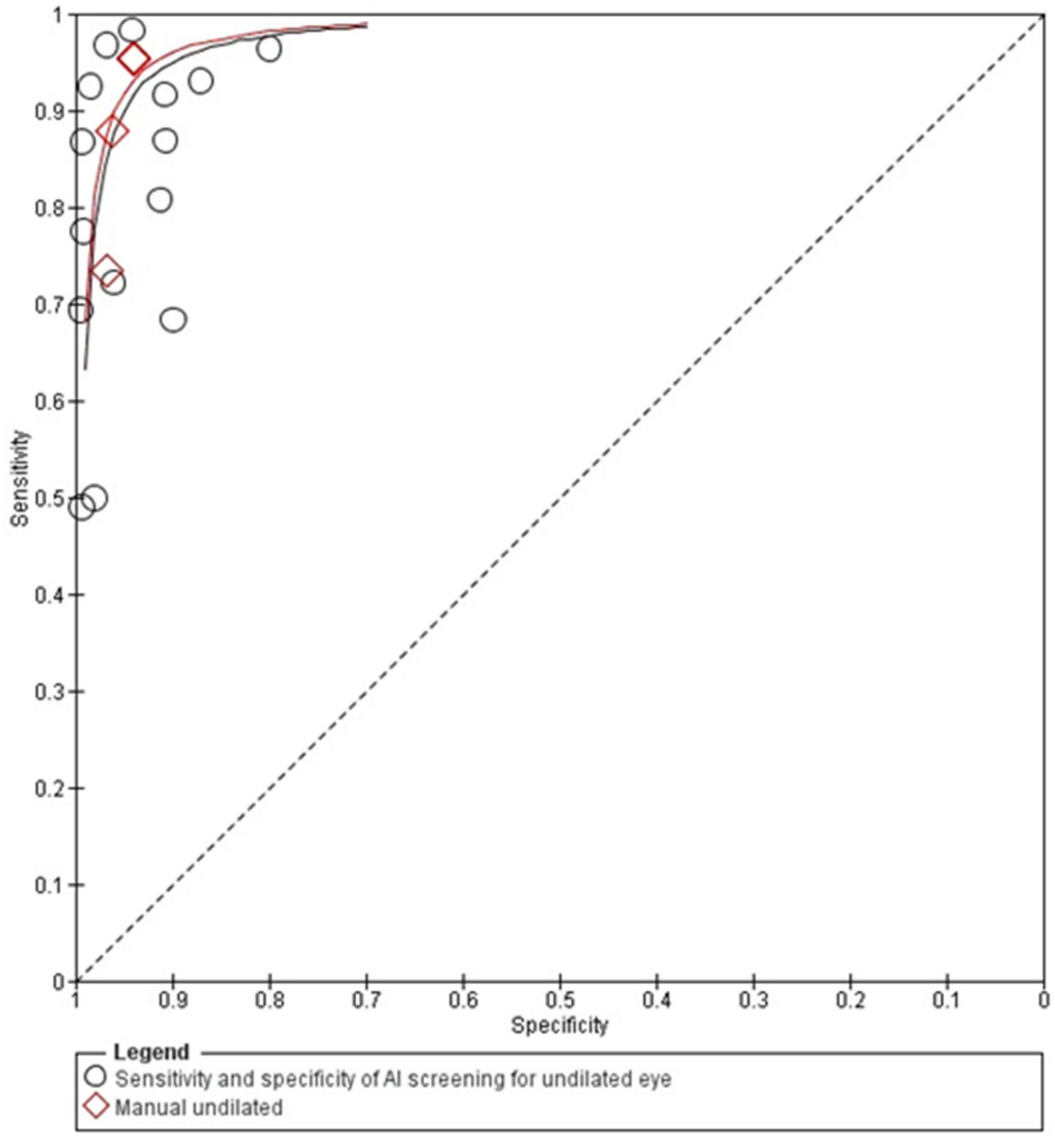
SROC plot for un-dilated eyes screening.

**Figure 3:**
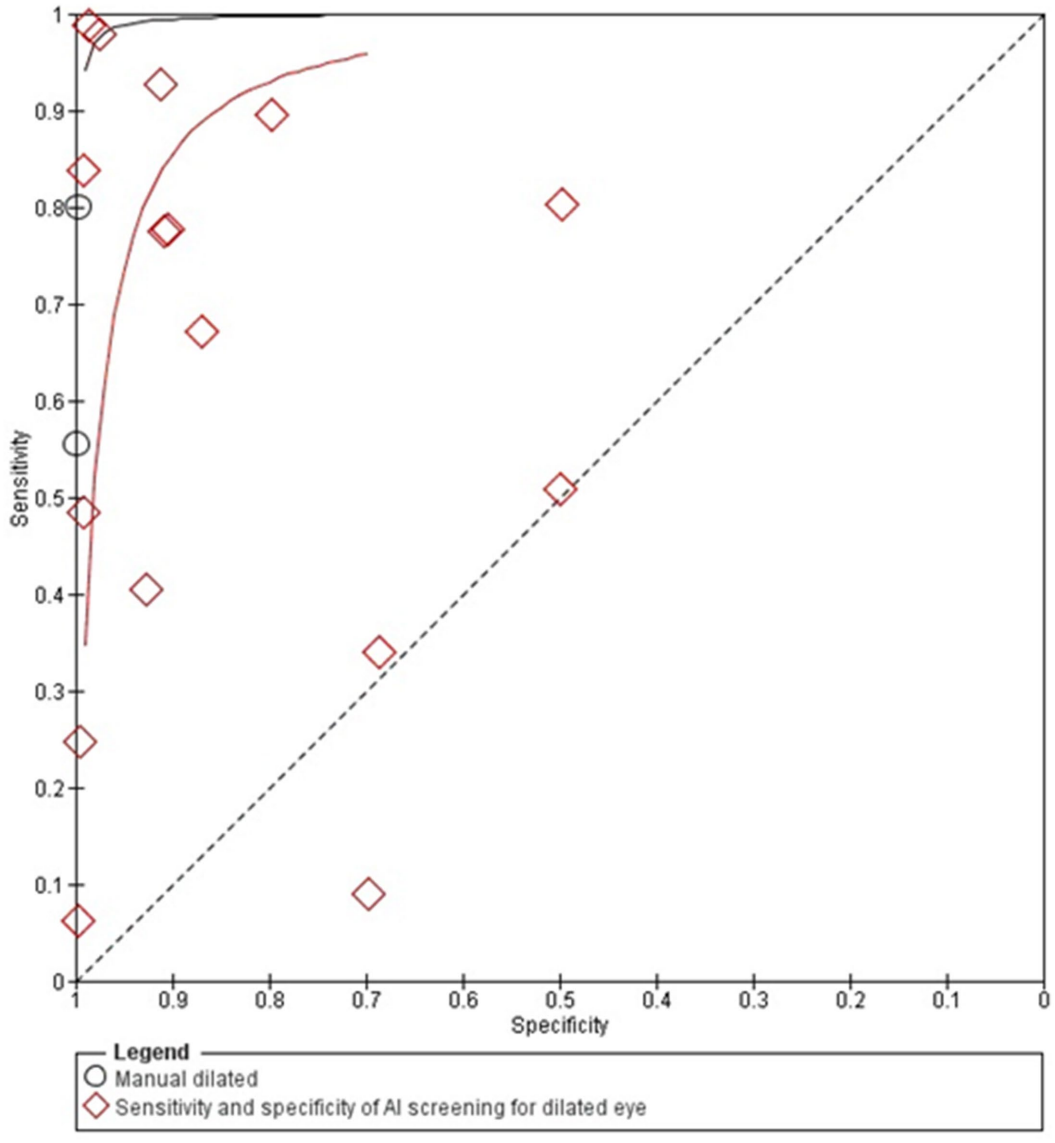
SROC plot for dilated eyes screening.

Overall, these SROC plots highlight that AI models demonstrate robust diagnostic accuracy for detecting diabetic retinopathy in both dilated and un-dilated settings, with higher sensitivity and closer specificity compared to manual screening methods in most of the studies as can be seen in the Figures 2, 3.

### Sensitivity

**Figure 4:**
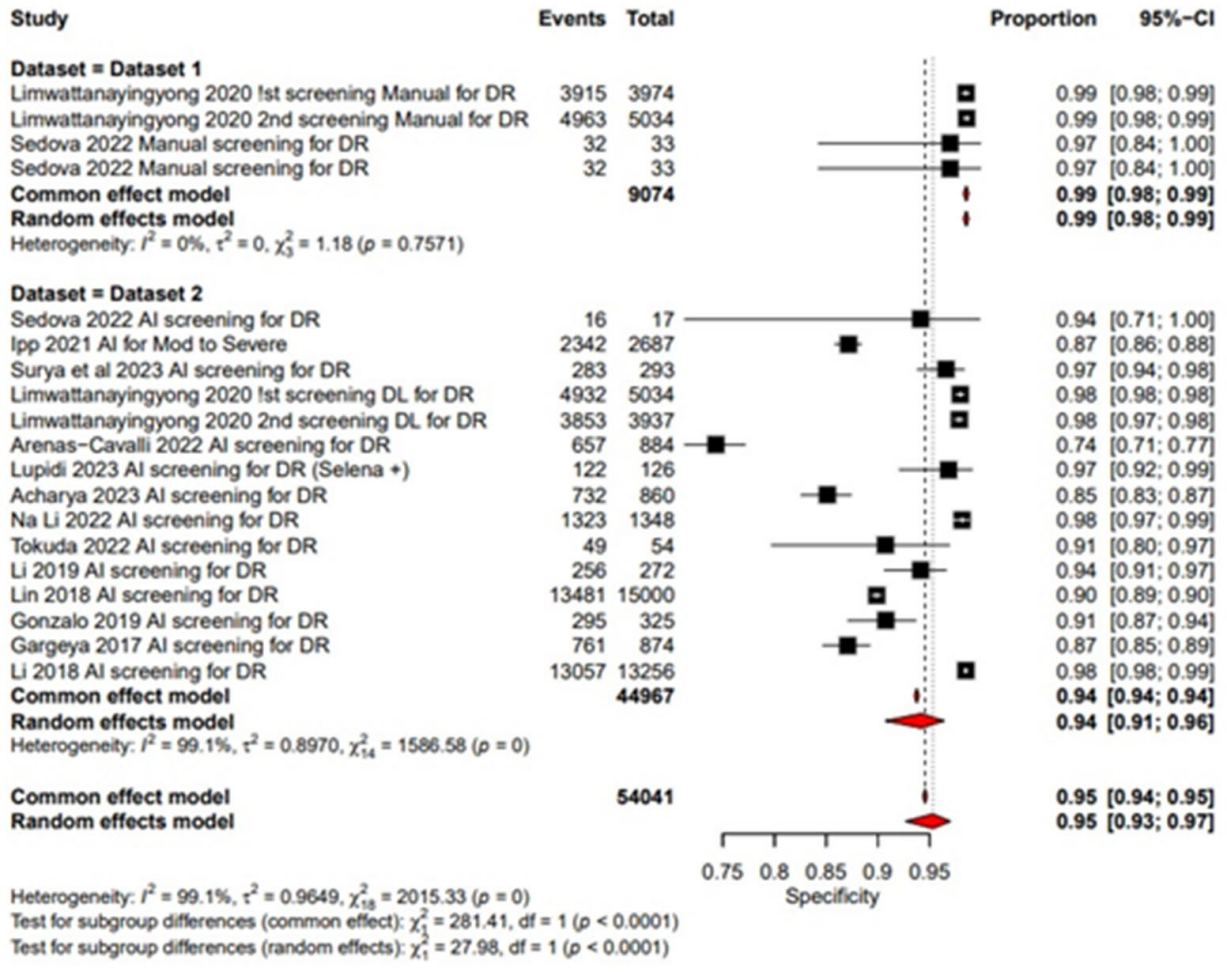
Specificity forest plot for un-dilated eyes.

**Figure 5:**
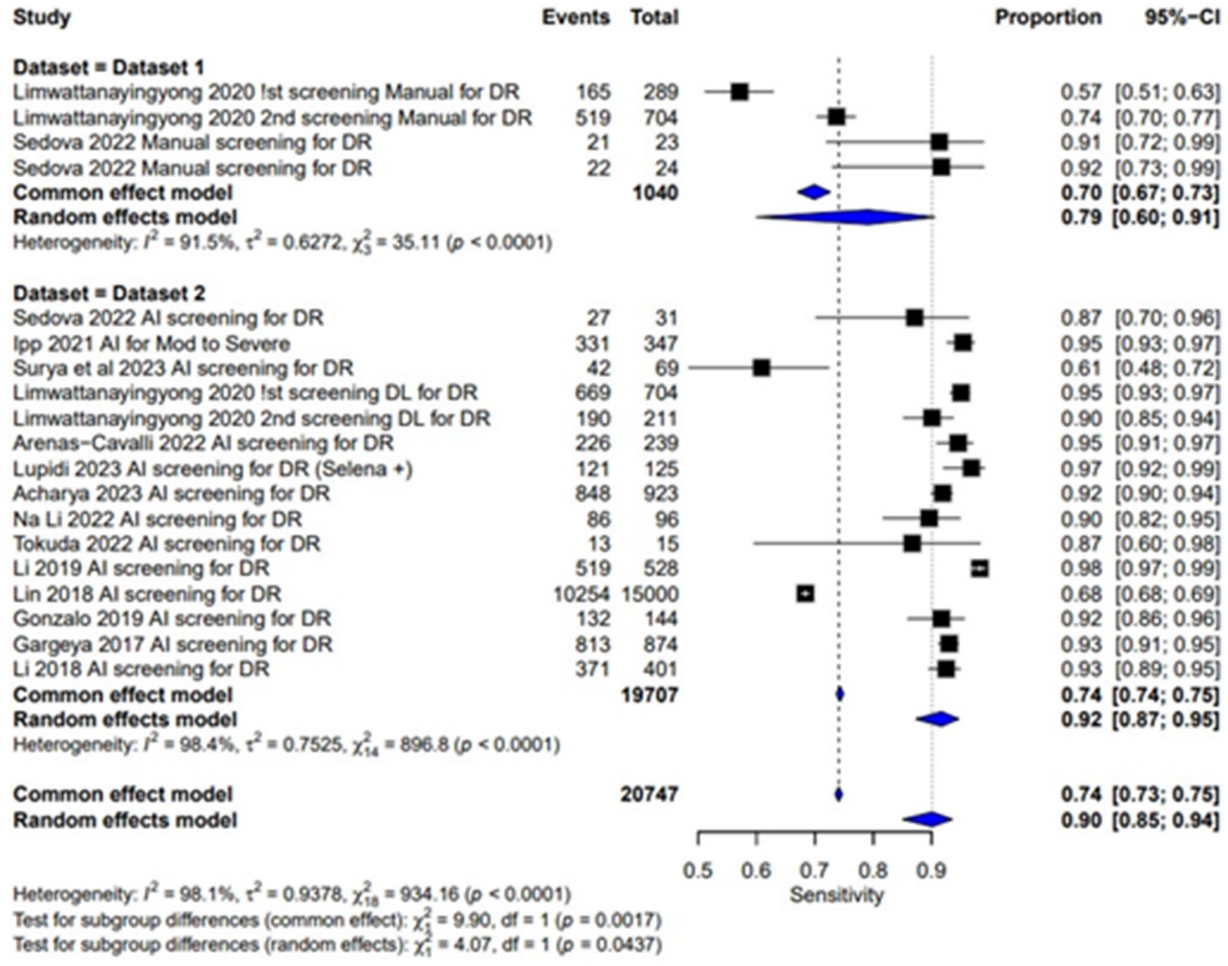
Sensitivity forest plot for un-dilated eyes.

The sensitivity of AI-based screening for dilated eyes show consistent results across the studies with a pooled sensitivity of 0.95 (95% CI: 0.91, 0.97). For manual screening in dilated eyes, the pooled sensitivity reported was 0.90 (95% CI: 0.87, 0.92), showing lower performance than AI as given in Table 2 and Figure 4. For un-dilated eyes, AI screening achieved a pooled sensitivity of 0.92 (95% CI: 0.87, 0.95). In the manual screening of un-dilated eyes images pooled sensitivities of 0.79 (95% CI: 0.60, 0.91) is reported given in Table 2 and Figure 5. AI-based screening shows higher performance than manual screening.

**Table 2:** Results for outcomes.

Study	Outcome	Dilated/Un-dilated eye	TP	FP	FP	TN	Sensitivity (CI at 95%)	Specificity (CI at 95%)
Piatti et al. 2024 (13)	Mild DR with AI	Dilated	70	102	102	399	0.41 [0.33, 0.48]	0.93 [0.90, 0.95]
Piatti et al. 2024 (13)	Moderate and beyond with AI	Dilated	35	0	0	0	1.00 [0.90, 1.00]	Not estimable
Sosale et al. 2020 (15)	AI for referable DR	Dilated	120	23	23	153	0.84 [0.77, 0.90]	0.99 [0.96, 1.00]
Sosale et al. 2020 (15)	AI for any DR	Dilated	105	8	8	168	0.93 [0.87, 0.97]	0.91 [0.86, 0.95]
Ting et al. 2017 (25)	AI for referable DR	Dilated	3,057	9,172	9,172	100,097	0.25 [0.24, 0.26]	1.00 [1.00, 1.00]
Ting et al. 2017 (25)	Moderate and beyond with AI	Dilated	676	9,969	9,969	102,003	0.06 [0.06, 0.07]	1.00 [1.00, 1.00]
Ipp 2021 (10)	AI for Mod and beyond	Dilated	356	375	375	2,630	0.49 [0.45, 0.52]	0.99 [0.99, 1.00]
Soto-Pedre et al. 2015 (18)	AI screening for DR	Dilated	535	1,034	1,034	2,277	0.34 [0.32, 0.37]	0.69 [0.67, 0.70]
Wang et al. 2018 (20)	AI screening for DR	Dilated	213	205	205	206	0.51 [0.46, 0.56]	0.50 [0.45, 0.55]
Abràmoff et al. 2016 (31)	AI screening for DR	Dilated	182	88	88	598	0.67 [0.61, 0.73]	0.87 [0.84, 0.90]
Hansen et al. 2015 (29)	AI screening for DR	Dilated	91	900	900	2,093	0.09 [0.07, 0.11]	0.70 [0.68, 0.72]
Rajalakshmi et al. 2018 (19)	AI screening for DR	Dilated	184	21	21	84	0.90 [0.85, 0.94]	0.80 [0.71, 0.87]
Kumar et al. 2016 (34)	AI screening for DR	Dilated	722	176	176	176	0.80 [0.78, 0.83]	0.50 [0.45, 0.55]
Zhang et al. 2019 (32)	AI screening for DR (Grading system)	Dilated	414	4	4	344	0.99 [0.98, 1.00]	0.99 [0.97, 1.00]
Zhang et al. 2019 (32)	AI screening for DR (identification system)	Dilated	412	8	8	340	0.98 [0.96, 0.99]	0.98 [0.96, 0.99]
Zhang et al. 2022 (33)	AI screening for DR (InceptionV3_299)	Dilated	12,440	3,580	3,580	35,953	0.78 [0.77, 0.78]	0.91 [0.91, 0.91]
Zhang et al. 2022 (33)	AI screening for DR (InceptionV3_896)	Dilated	12,984	3,676	3,676	35,857	0.78 [0.77, 0.79]	0.91 [0.90, 0.91]
Sedova et al. 2022 (14)	AI screening for DR	Undilated	27	1	1	16	0.96 [0.82, 1.00]	0.80 [0.56, 0.94]
Ipp 2021 (10)	AI for Mod to Severe	Undilated	331	345	345	2,342	0.49 [0.45, 0.53]	0.99 [0.99, 1.00]
Surya et al. 2023 (16)	AI screening for DR	Undilated	42	10	10	283	0.81 [0.67, 0.90]	0.91 [0.88, 0.94]
Limwattanayingyong et al. 2020 (24)	1st screening DL for DR	Undilated	669	102	102	4,932	0.87 [0.84, 0.89]	0.99 [0.99, 1.00]
Limwattanayingyong et al. 2020 (24)	2nd screening DL for DR	Undilated	190	84	84	3,853	0.69 [0.64, 0.75]	0.99 [0.99, 1.00]
Arenas-Cavalli et al. 2022 (23)	AI screening for DR	Undilated	226	227	227	657	0.50 [0.45, 0.55]	0.98 [0.97, 0.99]
Lupidi et al. 2023 (12)	AI screening for DR (Selena +)	Undilated	121	4	4	122	0.97 [0.92, 0.99]	0.97 [0.92, 0.99]
Acharyya et al. 2024 (22)	AI screening for DR	Undilated	848	128	128	732	0.87 [0.85, 0.89]	0.91 [0.88, 0.93]
Li et al. 2022 (11)	AI screening for DR	Undilated	86	25	25	1,323	0.77 [0.69, 0.85]	0.99 [0.99, 1.00]
Tokuda et al. 2022 (17)	AI screening for DR	Undilated	13	5	5	49	0.72 [0.47, 0.90]	0.96 [0.87, 1.00]
Li et al. 2019 (28)	AI screening for DR	Undilated	519	16	16	256	0.98 [0.97, 0.99]	0.94 [0.91, 0.97]
Lin et al. 2018 (27)	AI screening for DR	Undilated	10,254	1,519	1,519	13,481	0.68 [0.68, 0.69]	0.90 [0.89, 0.90]
González-Gonzalo et al. 2020 (26)	AI screening for DR	Undilated	132	30	30	295	0.92 [0.86, 0.96]	0.91 [0.87, 0.94]
Gargeya and Leng 2017 (30)	AI screening for DR	Undilated	813	113	113	761	0.93 [0.91, 0.95]	0.87 [0.85, 0.89]
Li et al. 2018 (21)	AI screening for DR	Undilated	371	199	199	13,057	0.93 [0.89, 0.95]	0.98 [0.98, 0.99]
Limwattanayingyong et al. 2020 (24)	!st screening Manual for DR	Undilated	165	124	59	3,915	0.74 [0.67, 0.79]	0.97 [0.96, 0.97]
Limwattanayingyong et al. 2020 (24)	2nd screening Manual for DR	Undilated	519	185	71	4,963	0.88 [0.85, 0.90]	0.96 [0.96, 0.97]
Sedova et al. 2022 (14)	Manual screening for DR	Undilated	21	2	1	32	0.95 [0.77, 1.00]	0.94 [0.80, 0.99]
Sedova et al. 2022 (14)	Manual screening for DR	Undilated	22	2	1	32	0.96 [0.78, 1.00]	0.94 [0.80, 0.99]
Ting et al. 2017 (25)	Manual for referable DR	Dilated	3,077	302	768	108,501	0.80 [0.79, 0.81]	1.00 [1.00, 1.00]
Ting et al. 2017 (25)	Moderate and beyond with Manual	Dilated	558	78	447	111,525	0.56 [0.52, 0.59]	1.00 [1.00, 1.00]

CI, Confidence Interval; DR, Diabetic Retinopathy; Referable DR, severity grade 2 and above; DL, Deep Learning; DLA, Deep Learning Algorithm; FN, False Negative; FP, False Positive; Mod, Moderate; RDR, Referable Diabetic Retinopathy; SVM, Support Vector Machine; TP, True Positive; TN, True Negative; UWF, Ultra-Wide Field Grading.

### Specificity

**Figure 6:**
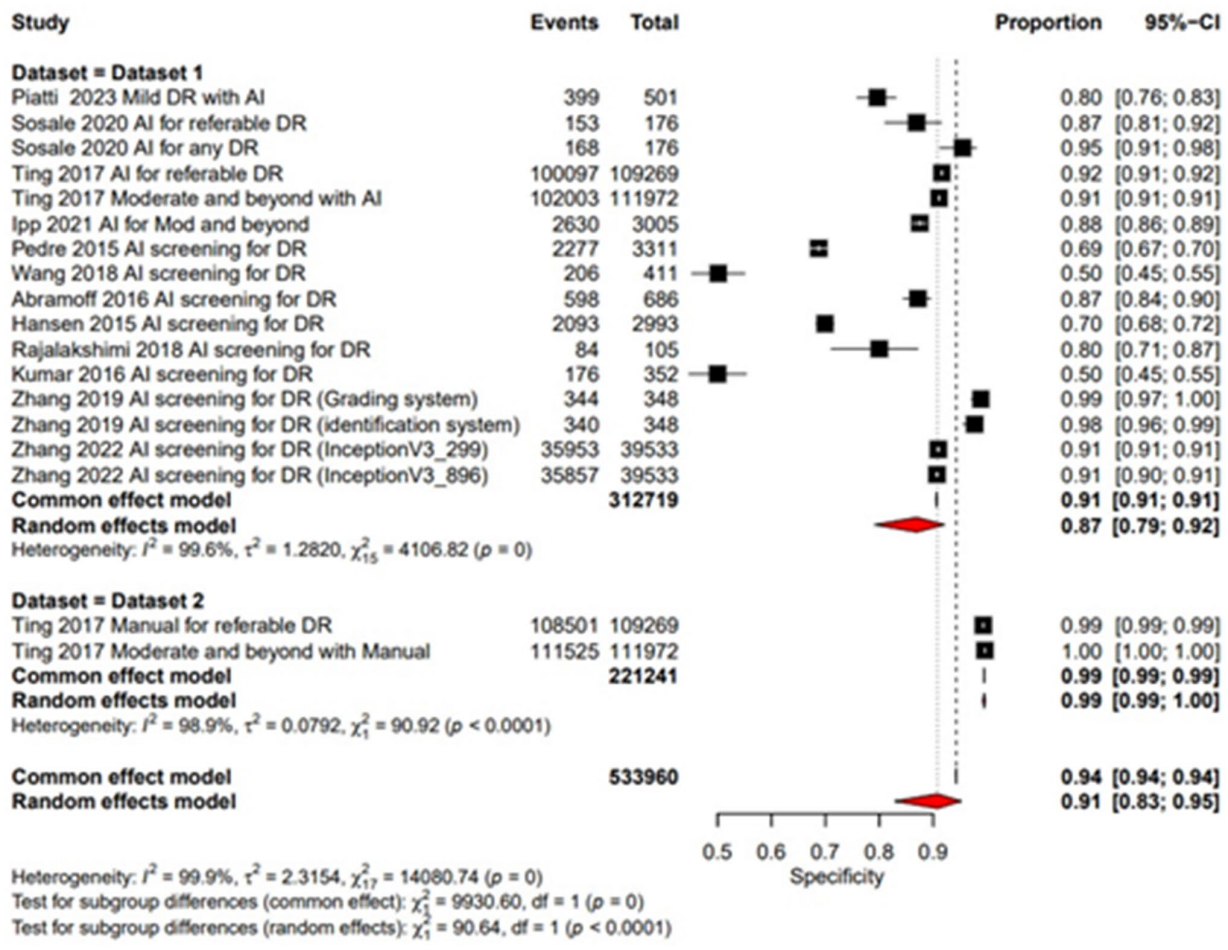
Specificity forest plot for dilated eyes.

**Figure 7:**
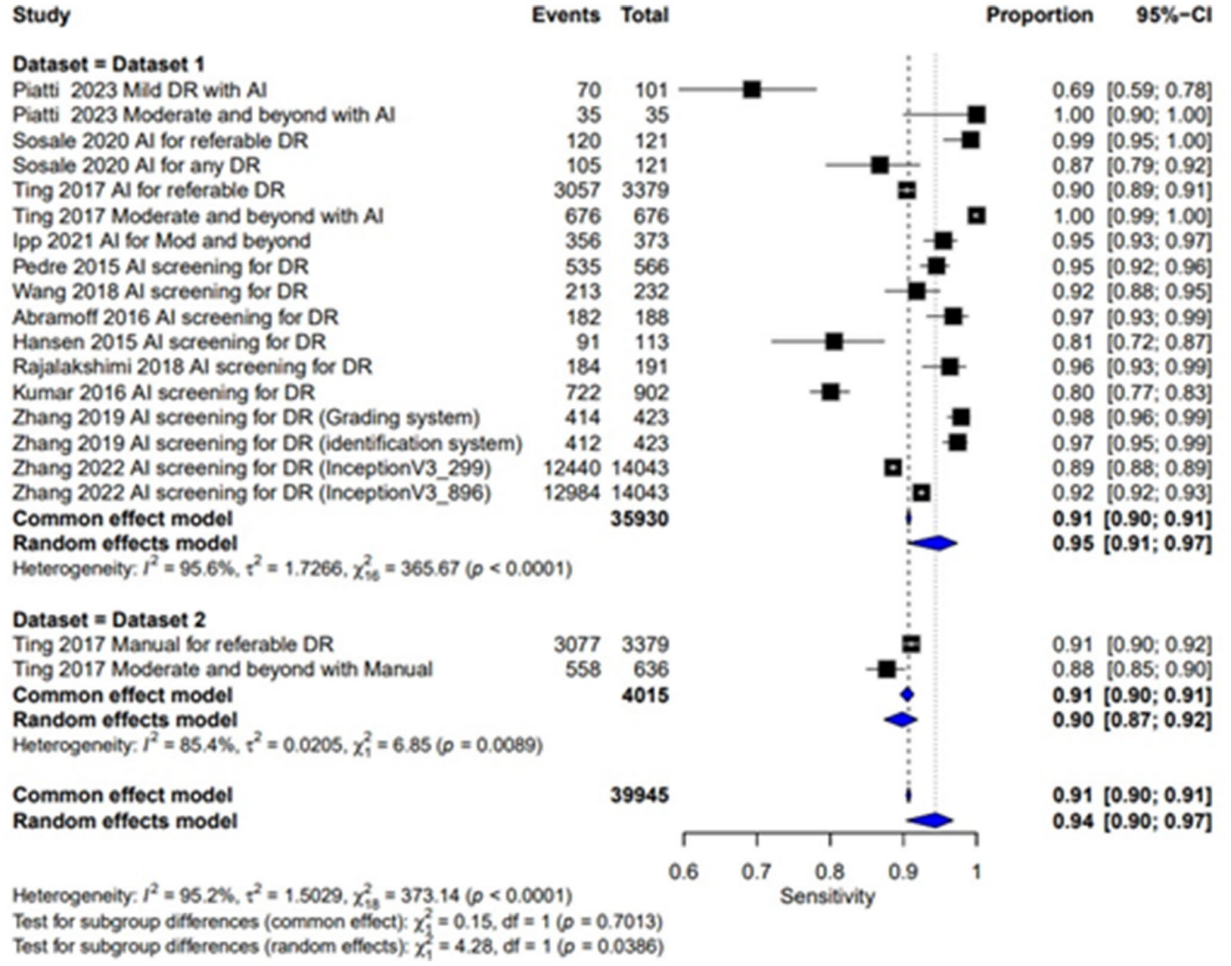
Sensitivity forest plot for dilated eyes.

Pooled specificity of AI screening for dilated eyes was reported at 0.87 (95% CI: 0.79, 0.92) showing a good performance and manual screening for dilated eyes also showed a high pooled specificity value of 0.99 (95% CI: 0.99, 1.00). Showing a good performance of both AI-based and manual screening methods as shown in the Figure 6. For un-dilated eyes, AI screening demonstrated pooled specificity of 0.94 (95% CI: 0.91, 0.96). Manual screening similarly showed robust specificity 0.99 (95% CI: 0.98, 0.99) as given in the Figure 7. Showing that AI a comparable alternative to manual screening.

### Multi-test analysis

The combined pooled sensitivity and specificity of dilated eye is 0.94 [95% CI: 0.90; 0.97] and 0.91 [0.83; 0.95] with heterogeneity of 95.2 and 99.9% and *p* value of 0.0386 and 0.0001, respectively, showing comparable results in the outcomes with high variability among studies as shown in Figures 4, 6. Un-dilated eye report combined pooled sensitivity and specificity of 0.90 [95% CI: 0.85; 0.94] and 0.95 [0.93; 0.97] with heterogeneity of 98.1 and 99.1% and *p* value of 0.0437 and 0.0001, respectively, showing results with no statistically significant difference as shown in Figures 5, 7.

### Risk of bias

**Figure 8:**
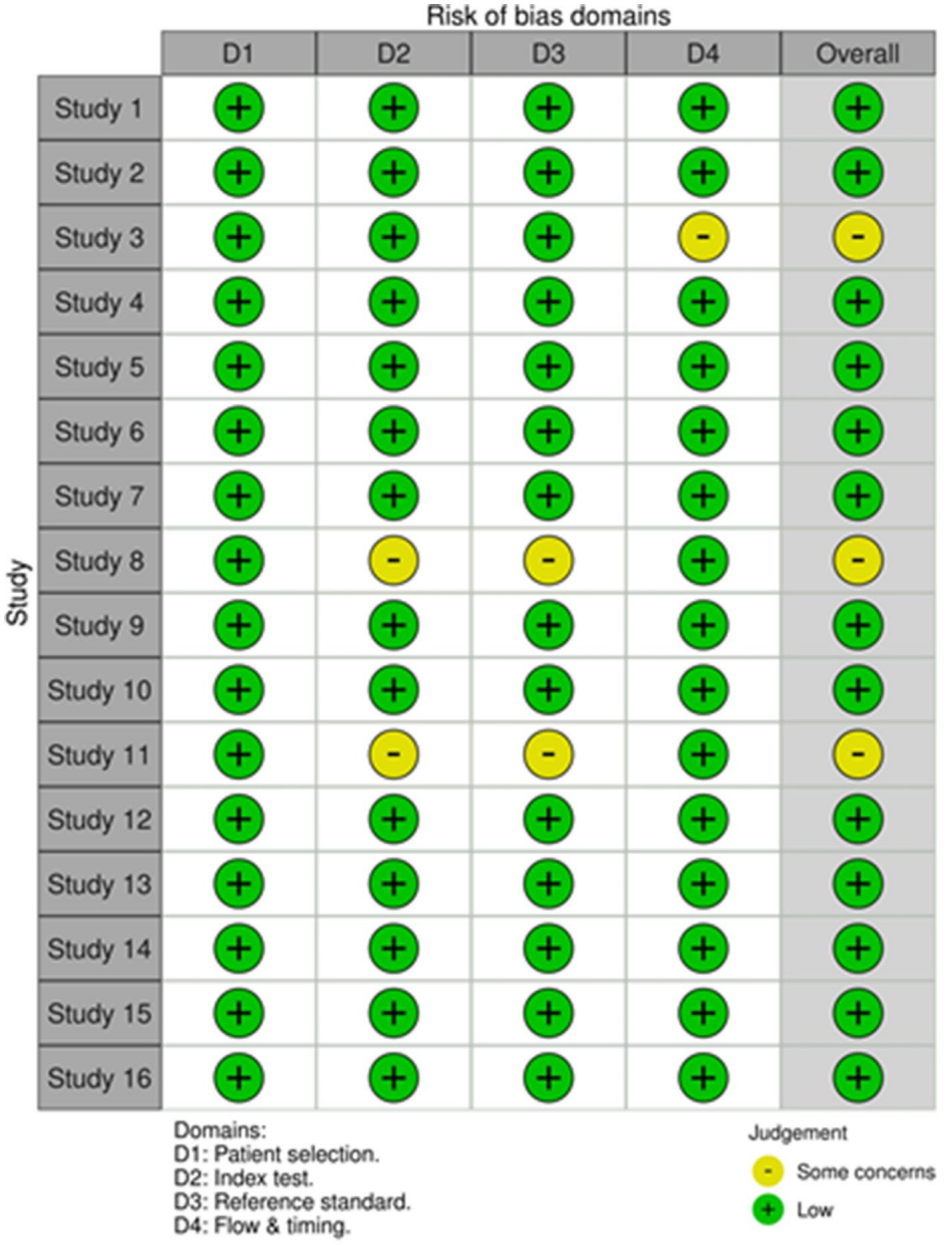
Risk of bias assessment traffic light plot for QUADAS-2 tool.

**Figure 9:**
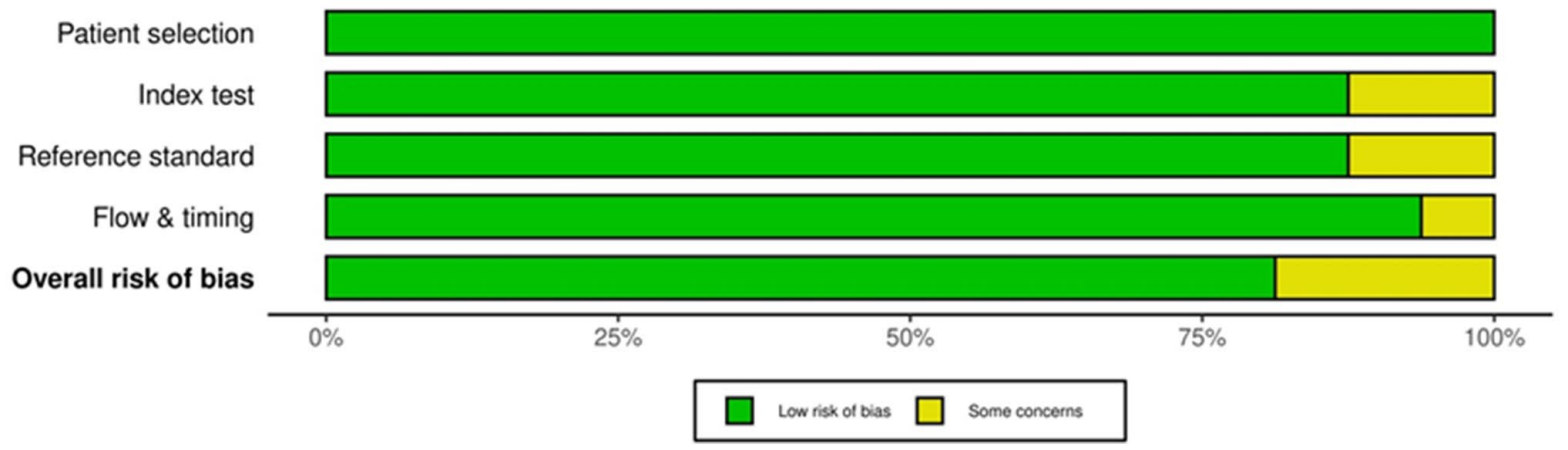
Risk of bias assessment summary plot for QUADAS-2 tool.

Risk of bias was systematically assessed using appropriate tools for the study designs. For the 16 validation studies (13, 16, 18–21, 23, 26–34), the QUADAS-2 tool was used. Thirteen of these studies demonstrated a low risk of bias, while three study shows some concerns particularly in the domain 3 and 4, as shown in the accompanying Figures 8, 9.

For the five cross-sectional studies, the AXIS tool was used to assess the risk of bias (10, 12, 15, 22, 25). The results reported a moderate risk of bias across the studies, with bias related to results and conclusion. These findings are summarized in Table 3.

**Table 3:** AXIS risk of bias assessment summary-percentages of items satisfied.

Author	Intro	Methods	Results	Conclusions	Other	Risk
Ting et al. 2017 (25)	100%	100%	50%	75%	50%	Moderate
Sosale et al. 2020 (15)	100%	100%	50%	75%	0%	Moderate
Ipp 2021 (10)	100%	100%	50%	50%	100%	Moderate
Acharyya et al. 2024 (22)	100%	90%	50%	75%	0%	Moderate
Lupidi et al. 2023 (12)	100%	100%	50%	75%	0%	Moderate

AXIS, Appraisal tool for Cross-Sectional Studies; %, percentage of the bias.

In the risk of bias assessment of four cohort studies, the Newcastle-Ottawa Scale was applied. All four studies demonstrated a low risk of bias, in all domains such as selection, comparability, and outcome assessment (11, 14, 17, 24). These results are detailed in Table 4, supporting the reliability of the included cohort studies.

**Table 4:** Asterisk rating in observational studies according Newcastle-Ottawa scale (NOS) tool.

Study	Adequacy of selection				Comparability	Outcome assessment			Asterisk rating	Overall
	Representative of the exposed cohorts	Selection of the exposed cohorts	ascertainment of exposure	Demonstration that Outcome of Interest was Not Present at Start of Study		Assessment of outcomes	Follow-up period long enough for outcome to occur	Adequacy of follow-up period among cohorts		
Sedova et al. 2022 (14)	*		*	*	**	*	*	*	8.0/9.0	Low
Tokuda et al. 2022 (17)	*		*	*	**	*		*	7.0/9.0	Low
Li et al. 2022 (11)	*		*	*	**	*		*	7.0/9.0	Low
Limwattanayingyong et al. 2020 (24)	*		*		**	*	*	*	7.0/9.0	Low

NOS, Newcastle-Ottawa Scale; **Indicates two stars in NOS for comparability domain; *Indicates one star for selection, comparability, or outcome assessment based on NOS guidelines for cohort studies; Adequacy of follow-up period: evaluated based on sufficient follow-up time.

## Discussion

The development of artificial intelligence based screening systems has led to potential use as a diagnostic tool in health care system. Evaluating the accuracy of AI in clinical settings is essential to ensure its implementation in clinical settings. Diabetic retinopathy screening is important in preventing vision loss. In this meta-analysis, we assessed the diagnostic accuracy of AI-based systems versus manual screening methods for both dilated and un-dilated eyes, for detecting DR. The aim was to determine whether AI systems could offer a comparable or superior alternative to manual methods in clinical practice.

Our results showed that AI systems demonstrated a high sensitivity across most studies. In comparison sensitivity for both dilated and un-dilated eyes using AI screening shows a good performance and specificity for AI screening and manual screening was generally comparable, with dilated eyes as well as un-dilated eyes.

These results highlight that AI systems, especially in un-dilated eye conditions, show promise for clinical use with reliable sensitivity and specificity, but variations exist depending on the system and clinical setting.

Most of the studies exhibit low risk of bias showing which shows robust methodologies and reliable findings but some validation studies have shown moderate risk of bias especially in index test and reference standards suggesting possible inconsistencies in diagnostic criteria or lack of blinding. Also the studies assessed with axis tool shows moderate risk of bias in all studies especially in the results and conclusion domain indicates potential selective reporting, which could introduce bias in outcome interpretation.

### Limitations and implications

Despite the promising outcomes, several limitations must be acknowledged. First, there is considerable heterogeneity across the included studies in terms of study settings, photographic protocols, and reference standards. The studies vary from community-based to outpatient settings, and the imaging techniques range from two-field to five-field photography with or without mydriasis. These differences may have influenced the diagnostic performance of AI based screening, limiting the generalizability of the findings. Additionally, the reference standards used for manual grading differ across studies, with some having single specialists and others using diagnoses by multiple experts, potentially affecting the accuracy of comparisons. Second, not all studies report the number of participants, making it difficult to assess the true sample size, which could impact diagnostic validity. Third, there is a significant variability among the studies in AI based screening, Variability in AI performance can arise from differences in study methodologies, dataset quality, and model training conditions. The findings highlight the need for standardized evaluation metrics and more transparent reporting to solve inconsistencies. Addressing these issues will enhance the reliability of AI applications in clinical settings and ensure robust decision-making.

Moreover, some of the studies had a moderate risk of bias which could lead to over-estimation or down-estimation of accuracy. To ensure that AI systems are safe and effective for real-world use, evaluations need to be conducted in representative clinical settings. Systems should be tested on a wide range of image qualities, and medical settings.

## Conclusion

The findings from this meta-analysis suggest that AI systems are promising for DR screening, especially in settings where high sensitivity is critical. However, further independent studies, particularly those assessing the dilated eyes screening, are required to establish the efficacy of AI in broader clinical practice. Factors such as system technical failures, and operational settings should also be considered before full implementation. In conclusion, while AI-based systems offer a valuable tool for reducing the workload on human graders, their clinical utility depends on continued rigorous evaluation and refinement.

### Future research

Future work should focus on refining AI algorithms for dilated eye conditions and exploring the integration of AI screening into routine ophthalmic practice. Large-scale, prospective validation studies will be essential to confirm these findings and guide the adoption of AI in DR screening protocols.

## Data Availability

The raw data supporting the conclusions of this article will be made available by the authors, without undue reservation.
